# Diagnostic and Therapeutic Challenges in Severe Peristomal Pyoderma Gangrenosum

**DOI:** 10.1093/ibd/izae167

**Published:** 2024-07-19

**Authors:** Bernadett Farkas, Emese Ivány, Anita Bálint, Peter Bacsur, Tamás Molnár, Klaudia Farkas

**Affiliations:** Center for Gastroenterology, Department of Internal Medicine, University of Szeged, Szeged, Hungary; Center for Gastroenterology, Department of Internal Medicine, University of Szeged, Szeged, Hungary; Center for Gastroenterology, Department of Internal Medicine, University of Szeged, Szeged, Hungary; Center for Gastroenterology, Department of Internal Medicine, University of Szeged, Szeged, Hungary; Center for Gastroenterology, Department of Internal Medicine, University of Szeged, Szeged, Hungary; Center for Gastroenterology, Department of Internal Medicine, University of Szeged, Szeged, Hungary; HCEMM-USZ Translational Colorectal Research Group, University of Szeged, Szeged, Hungary

## Background

Ustekinumab (UST) targets the p40 subunit of interleukin-12/23. Because of its mechanism of action, UST may also be suitable for the treatment of pyoderma gangrenosum (PG).^1,2^ Data on the treatment of ulcerative colitis (UC)–associated PG with UST are limited.^3,4^

**Figure 1.**
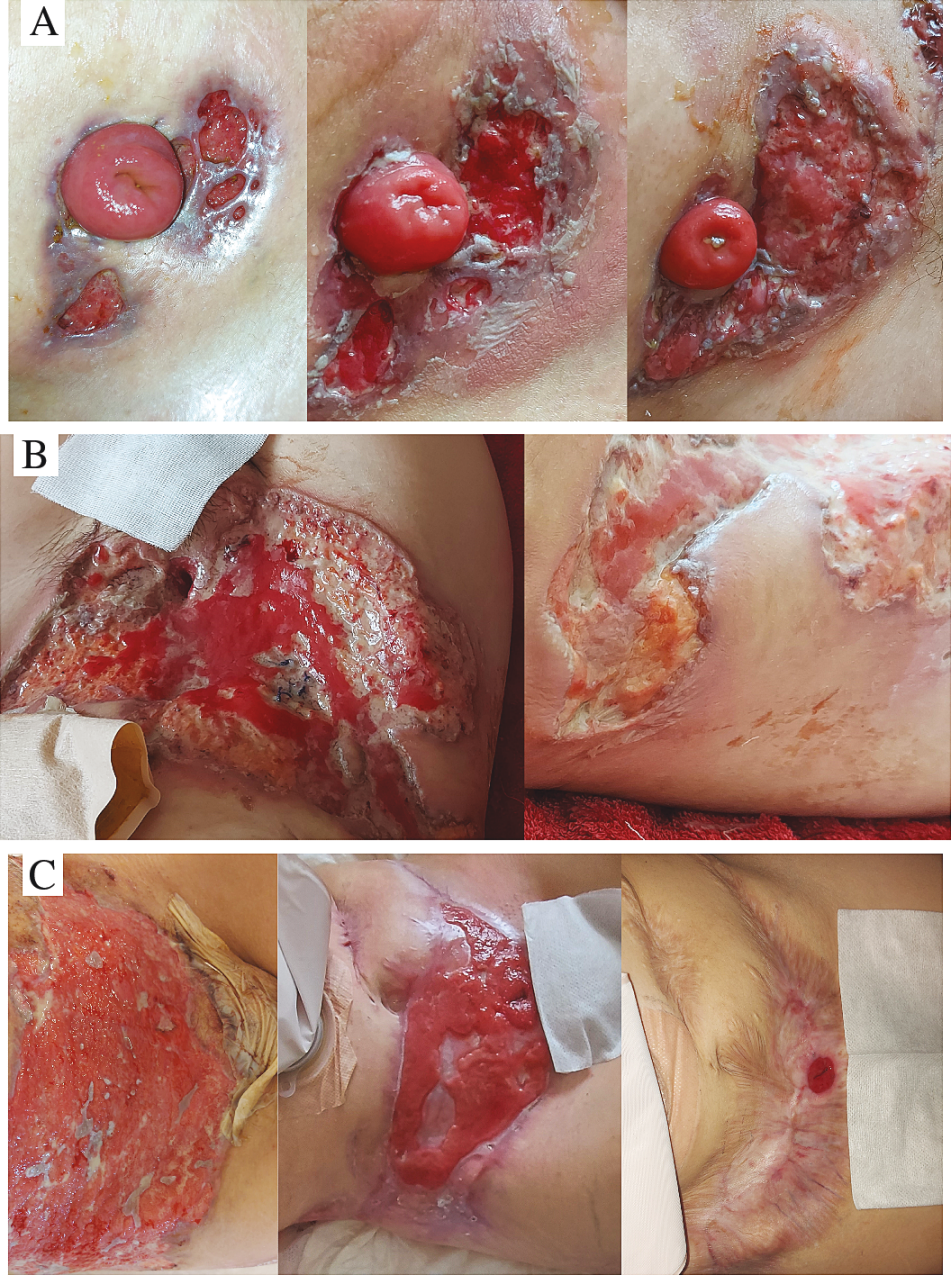
(A) The progression of peristomal pyoderma gangrenosum with β-lactam antibiotic therapy. (B) The isolated skin lesions had merged into one penetrating ulcer measuring approximately 37 × 15 cm. To resolve the constant maceration and contamination by fecal matter, the ileostoma was repositioned to the left upper abdomen. (C) The appearance of peristomal pyoderma gangrenosum during the course of ustekinumab treatment.

A 31-year-old female UC patient was started on infliximab in October 2022. The first induction was followed by a severe relapse. The new-onset axillary hidradenitis suppurativa was successfully treated with doxycycline. Due to steroid-refractory UC, cyclosporine was initiated in combination with vedolizumab. Nevertheless, she underwent subtotal colectomy in mid-November 2022. After discharge, peristomal, livid-edged ulcers of 5 to 30 mm in size appeared on the patient (**Figure 1A**). Cefuroxime was started for 7 days, followed by amoxicillin-clavulanic acid for 14 days. Despite targeted antibiotic therapy, the isolated skin lesions had merged into one penetrating ulcer measuring approximately 37 × 15 cm. To resolve the constant maceration and contamination by fecal matter, the ileostoma was repositioned to the left upper abdomen, and negative pressure wound therapy was used to facilitate wound healing (**Figure 1B**). As the wound base was cleared, skin transplantation was performed with partial-thickness grafts, as recommended by plastic surgeons. Following the failure of graft adhesion, a dermatology consultation was arranged, and PG was diagnosed based on the following findings: rapidly progressing disease, history of UC, pathergy, peripheral erythema with violaceous borders, poor response to antibiotics, the exclusion of other causes, and neutrophilic inflammation in histopathology. Intravenous methylprednisolone (1 mg/kg) was started with UST (390 mg intravenous). With 4 times weekly UST and slow tapering of the corticosteroid (reduced by 4 mg every week to 8 mg, then discontinued after 3 months), the PG lesions were completely healed 10 months later (**Figure 1C**).

The diagnosis of peristomal PG is challenging. Because no validated diagnostic criteria for PG exist, the diagnosis is based on the combination of a comprehensive clinical assessment and the exclusion of other causes of skin ulceration.^5^ Nonhealing ulcers require dermatology consultation. Inadequate treatment of PG can lead to life-threatening complications and permanent impairment.

UST is currently not approved for use in PG. In the present case, the administration of UST was based on the good results reported in previous case series and retrospective studies on PG.^6-8^ Furthermore, UST was used to achieve sustained remission after urgent subtotal colectomy for refractory UC.

Our case suggests that UST might be an effective and safe therapeutic option for peristomal PG. Further studies are needed to confirm our findings.

## Data Availability

This case report details anonymized data of the patient, which was collected and presented with the patient’s written informed consent, in line with standards of publication ethics as outlined by the Commission on Publication Ethics guidelines.
